# Thyroid function and thyroid antibodies in patients with alopecia areata: a comparison of clinical patterns^⋆^

**DOI:** 10.1016/j.abd.2022.10.007

**Published:** 2023-03-30

**Authors:** Winnie Celorio, Luisa Cifuentes, Erika Cantor, Adriana Wagner

**Affiliations:** a Dermatology Programme, Universidad Libre Seccional Cali, Cali, Colombia; b Department of Dermatology, Clínica Imbanaco Grupo Quirón Salud, Cali, Colombia; c Institute of Statistics, Universidad de Valparaíso, Valparaíso, Chile

Dear Editor,

Alopecia areata (AA) is a disease that affects both men and women, with an overall prevalence of 1.2% to 2.2%.^1^, ^2^ A strong connection has been reported between AA and thyroid gland diseases, this is because both diseases share a common autoimmune component with similar genetic variations.^1^ Several case-control studies have reported a higher proportion of cases with a diagnosis of thyroid diseases or with altered parameters in thyroid function and autoantibody tests, as compared to the general population.^3^, ^4^ However, the studies analyzing this association according to the clinical presentations of AA (e.g., diffuse AA, multilocular AA, and ophiasis AA) are still limited.

The objective of this study was to compare, according to the clinical patterns of AA, the result variations in thyroid function tests (Thyroxine ‒ T4 and thyroid stimulating hormone - TSH) and in thyroid antibody tests (Thyroglobulin Antibody ‒ TGAb and Thyroid Peroxidase Antibody ‒ TPOAb) in a cohort of patients that were screened for thyroid abnormalities during the diagnostic process of AA in a Latin American center.

We performed a retrospective review of cases with AA diagnosed between 2017 and 2020 in Cali, Colombia. Adults identified with a clinical pattern compatible with diffuse AA, multilocular AA, or ophiasis AA, who underwent thyroid function screening during the diagnosis of AA were analyzed. Only patients who had at least one measurement of T4 or TSH and TGAb or TPOAb were included in this report. Ethical approval was obtained from the Institutional review board.

T4 or TSH values outside the normal range were classified as abnormal thyroid function. Similarly, an altered thyroid antibody level was defined by measurements of TPOAb or TGAb. The normal cut-off ranges were 5.0–11.0 ug/dL for T4, 0.4–4.0 IU/mL for TSH, less than 35 IU/mL for TPOAb, and less than 20 IU/mL for TgA. Cases with a previous diagnosis of thyroid disease were considered with abnormal thyroid function and altered antibody levels.

All analyses were carried out in Stata version 16.0 (StataCorp, Texas, USA). The Kruskal-Wallis nonparametric test was used for the comparison of quantitative variables. Qualitative variables were tested using the Chi-square or Fisher’s exact test. A p-value less than 0.05 was considered statistically significant.

A total of 89 patients with AA were included in this study, 32 (36.0%) of them had a diffuse pattern, 31 (34.8%) had a multilocular pattern, and 26 (29.2%) had an ophiasic pattern. The median age was 40 years (Interquartile Range ‒ IQR 35 to 52 years), and 85.4% (76) were women. Approximately one in four AA patients reported a concomitant diagnosis of thyroid pathology, and all were being treated with levothyroxine. The most frequent concomitant thyroid condition was hypothyroidism (n = 20), followed by thyroid nodules (n = 2) and cancer (n = 1) (Table 1).

**Table 1:** Clinical characteristics and thyroid dysfunction in cases with AA.

Variables	Total	Diffuse AA	Multilocular AA	Ophiasis AA	p-value
	(n = 89)	(n = 32)	(n = 31)	(n = 26)	
Age (years); Median (IQR)	40.0 (35.0‒52.0)	39.0 (32.2‒52.5)	43.0 (31.0‒53.0)	41.0 (35.0‒49.2)	0.883
Sex, n (%)					0.004
Female	76 (85.4)	30 (93.7)	21 (67.7)	25 (96.1)	
Male	13 (14.6)	2 (6.2)	10 (32.3)	1 (3.9)	
Symptom duration, months					0.105
No.	81	29	29	23	
Median (IQR)	8 (3‒21)	12 (5‒42)	5 (2‒12)	6 (3‒12)	
Comorbidities, n (%)					
Autoimmune disease	6 (6.7)	3 (9.4)	2 (6.4)	1 (3.8)	0.871
Thyroid disease	23 (25.8)	10 (31.2)	6 (19.3)	7 (26.9)	0.553
Altered T4					0.575
No.	77	28	27	22	
n (%)	6 (7.8)	1 (3.6)	3 (11.1)	2 (9.1)	
Altered TSH, n (%)	10 (11.2)	2 (6.2)	5 (16.1)	3 (11.5)	0.445
Abnormal thyroid function, n (%)	28 (31.5)	10 (31.2)	8 (25.8)	10 (38.5)	0.591
Altered TPOAb					0.528
No.	53	22	17	14	
Si, n (%)	11 (20.7)	3 (13.6)	4 (23.5)	4 (28.6)	
Altered TgA					0.392
No.	84	29	30	25	
Si, n (%)	19 (22.6)	6 (20.7)	5 (16.7)	8 (32.0)	
Altered thyroid antibody, n (%)	38 (42.7)	14 (43.7)	14 (45.2)	10 (38.5)	0.868
Thyroid dysfunction^a^ Si, n (%)	43 (48.3)	14 (43.7)	16 (51.6)	13 (50.0)	0.863

No., Number of non-missing observations; IQR, Interquartile Range; T4, Thyroxine T4; TSH, Thyroid Stimulating Hormone; TPOAb, Thyroid Peroxidase Antibody; TgAb, Thyroglobulin antibody.

a Patients with at least one altered parameter (T4, TSH, TPOAb and TgAb).

The proportion of cases with altered values on T4, TSH, TPOAb, or TGAb according to clinical AA patterns is shown in Table 1. The proportion of AA cases with altered antibody levels (42.7%) was higher than that with abnormal thyroid function (31.5%). In approximately half of AA cases, at least one abnormal thyroid result was reported as “Thyroid dysfunction” (Fig. 1). There were no statistically significant differences between cases with diffuse AA, multilocular AA, and ophiasis AA with respect to thyroid function and altered antibody levels (p > 0.05). Abnormal T4/TSH values were more common among patients with ophiasis AA (38.5%). Thyroid dysfunction was more common in cases with a multilocular and ophiasis pattern (≈50%).

**Figure 1:**
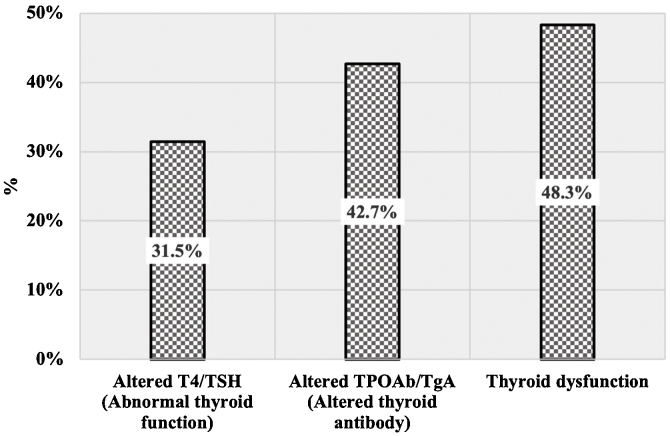
Proportion of AA cases with thyroid dysfunction.

Thus, the main finding of this study revealed that thyroid dysfunction is present in half of the patients with AA, without differences between the following patterns: diffuse, multilocular, and ophiasis. This estimate is higher than the reported rates for the general population (0.3% to 11.3%),^5^ suggesting a possible causal association between AA and thyroid dysfunction. However, the causal relationship remains unclear, with inconclusive results that give cause for further study. For example, in the recent study performed by Dai et al., a high risk of AA in patients with thyroid diseases and an increased risk of thyroid diseases in AA patients were reported, suggesting a bidirectional association between these conditions.^1^

Although the classification of AA cases according to clinical patterns is used to guide the treatment and prognosis in clinical practice, in the literature, it has been used infrequently to evaluate its association with thyroid abnormalities. In Egyptian patients with AA categorized as mild (3 patches with a diameter 3 cm), moderate (>3 patches with a diameter >3 cm), and severe (totalis or universalis AA), higher rates of abnormal values of TGAb (46% vs. 0%) and TPOAb (48% vs. 0%) were reported when compared to the healthy group. The highest mean of TSH, TGAb and TPOAb, as well as the lowest mean of T4 were reported in the severe group.^6^ Another study in which the AA patients were classified as mild-moderate and severe (totalis or universalis AA), described higher odds of hypothyroidism in patients with severe AA (Odds Ratio ‒OR = 1.74; 95% Confidence Interval ‒ 95% CI 1.387‒2.118).^7^

Due to the high prevalence of thyroid abnormalities in AA patients, initial screening tests have been recommended.^3^ Their use has been especially encouraged in severe AA, but this general recommendation remains controversial in mild and moderate forms.^8^ In this study, we included only cases with diffuse, multilocular, or ophiasis patterns representing a non-severe form of AA, to assess whether the performance of thyroid function screening should be prioritized with any of these patterns during clinical practice. Our results showed non-statistical differences in rates of thyroid dysfunction according to the AA pattern. Nevertheless, in ophiasis cases, the highest rate of abnormalities in thyroid function was reported, as well as a high rate of abnormal thyroid antibodies. These findings suggest that ophiasis cases may also constitute a high-risk group for thyroid disease. Furthermore, ophiasis AA is recognized as the form with the worst prognosis,^8^ therefore the detection and treatment of undiagnosed conditions associated with AA could help to achieve better outcomes.^9^

In conclusion, no statistically significant differences in rates of thyroid dysfunction according to clinical patterns were found. In this cohort, the incidence of abnormal thyroid function abnormalities was high; Therefore, it is recommended to perform thyroid function tests in all patients with AA at the time of diagnosis.

## Financial Support

None declared.

## Authors’ contributions

Winnie Celorio: Study conception and planning; critical literature review; data collection, analysis, and interpretation; preparation and writing of the manuscript; approval of the final version of the manuscript.

Luisa Cifuentes: Study conception and planning; critical literature review; preparation and writing of the manuscript; approval of the final version of the manuscript.

Erika Cantor: Study conception and planning; critical literature review; statistical analysis; data collection, analysis, and interpretation; preparation and writing of the manuscript; approval of the final version of the manuscript.

Adriana Wagner: Study conception and planning; critical literature review; effective participation in research orientation; preparation and writing of the manuscript; intellectual participation in propaedeutic and/or therapeutic management of studied cases; data collection, analysis, and interpretation; manuscript critical review; approval of the final version of the manuscript.

## Conflicts of interest

None declared.
